# Arbuscular mycorrhizal fungal contribution towards plant resilience to drought conditions

**DOI:** 10.3389/ffunb.2024.1355999

**Published:** 2024-02-16

**Authors:** Subhadeep Das, Soumyadev Sarkar

**Affiliations:** ^1^ Department of Biochemistry, Purdue University, West Lafayette, IN, United States; ^2^ Center for Fundamental and Applied Microbiomics, Biodesign Institute, Arizona State University, Tempe, AZ, United States

**Keywords:** plants, AMF, drought, symbiosis, mechanism

## Abstract

Climate changes cause altering rainfall patterns resulting in an increase in drought occurrences globally. These events are disrupting plants and agricultural productivity. To evade droughts, plants try to adapt and modify in the best capacities possible. The plants have adapted by structurally modifying roots, stems, and leaves, as well as modifying functions. Lately, the association of microbial communities with plants has also been proven to be an important factor in aiding resilience. The fungal representatives of the microbial community also help safeguard the plants against drought. We discuss how these fungi associate with plants and contribute to evading drought stress. We specifically focus on Arbuscular mycorrhizal fungi (AMF) mediated mechanisms involving antioxidant defenses, phytohormone mediations, osmotic adjustments, proline expressions, fungal water absorption and transport, morphological modifications, and photosynthesis. We believe understanding the mechanisms would help us to optimize the use of fungi in agricultural practices. That way we could better prepare the plants for the anticipated future drought events.

## 1 Introduction

**Figure 1:**
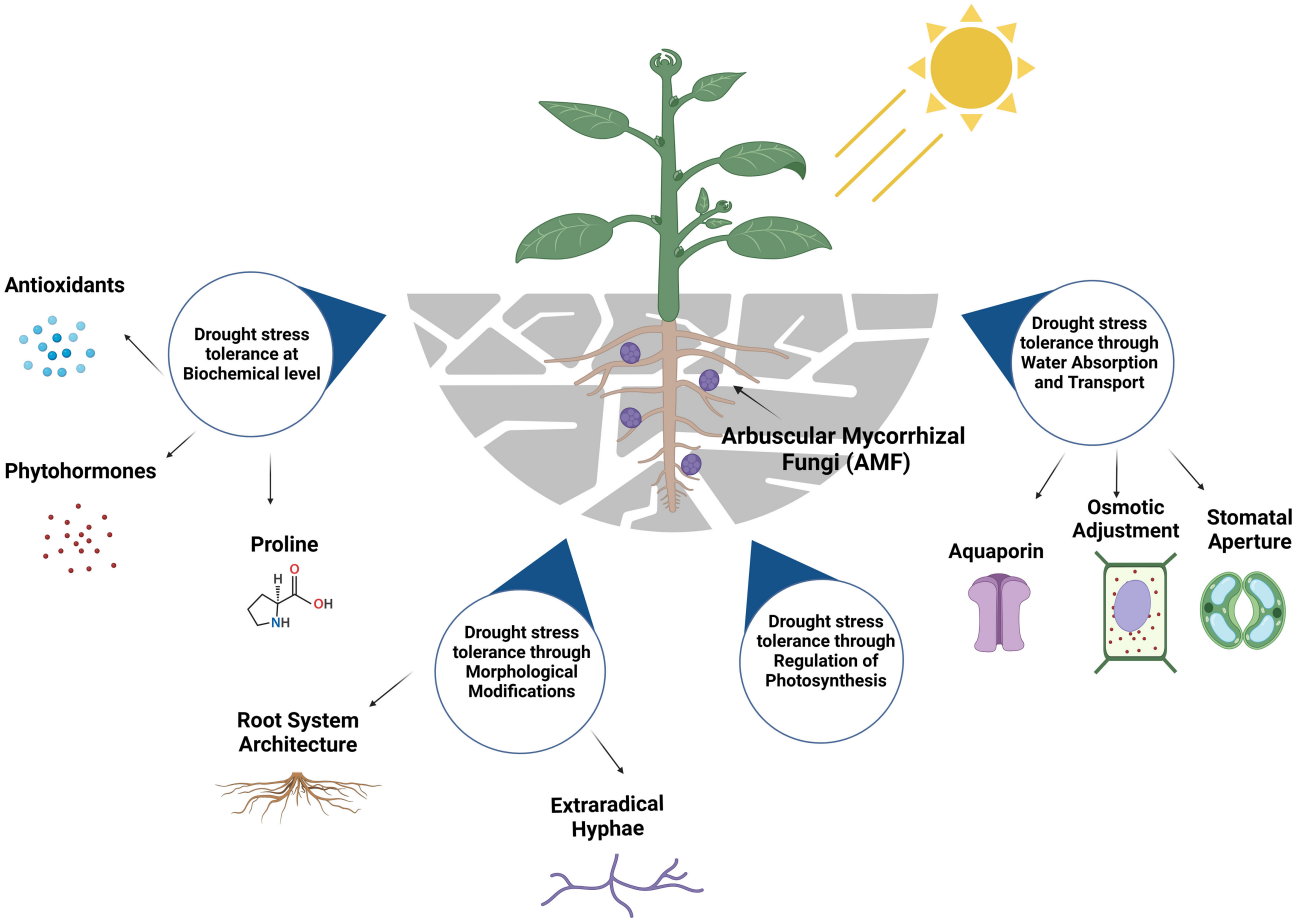
Key mechanisms for Arbuscular Mycorrhizal Fungi (AMF) to induce drought stress tolerance qualities into plants. The figure was created with BioRender.com.

Climate change is an immediate and global concern, as we anticipate more frequent drought events in the future (Leemans and Eickhout, 2004). Plants are expected to be directly affected by these drought events, impacting agricultural productivity (Backhaus et al., 2014; Feller and Vaseva, 2014; Mukherjee et al., 2018). Plants employ various strategies to cope with drought stress, enabling them to either evade stress or enhance their ability to tolerate drought. Plants can increase diffusive resistance, enhance water uptake by forming extensive root systems, and reduce transpiration loss, among others (Farooq et al., 2012). One of the other mechanisms enables plants to endure water-limited environments by sustaining a higher water status. Steadily, the knowledge about the involvement of the soil microbial communities aiding plants’ resilience is populating (Gamalero and Glick, 2011; Meena et al., 2017; Sarkar et al., 2022a, Sarkar et al., 2022b; Feng et al., 2023). Plants modify their microbiomes in response to various stressors, seeking assistance to cope with these challenges (Song and Haney, 2021). The role of yeast in environmental remediation is also well-known (Sarkar et al., 2019; Mukherjee et al., 2020; Ghosh et al., 2023; Rana et al., 2023; Vaksmaa et al., 2023). However, there is a dearth of understanding regarding the involvement of the fungal counterpart, particularly in terms of the mechanisms. Comprehending the role of soil fungi in bolstering plant resilience during drought conditions continues to be a formidable task, given the intricate nature of their composition and function (Emmett et al., 2021). Arbuscular mycorrhizal fungi (AMF) contribute to the resilience and adaptation of plants by withstanding environmental constraints, especially drought (Genre et al., 2020; Boczoń et al., 2021; Song and Haney, 2021; Abdalla et al., 2023; Cosme, 2023). AMF establishes a symbiotic association with around 80% of terrestrial plant species (Zobel and Öpik, 2014) enhancing plant tissue hydration and physiology during periods of drought stress (Ruiz-Lozano et al., 2012). It significantly affects plant growth, retention of water, mineral nutrition, as well as defense against abiotic stresses (Zhao et al., 2015). AMF plays a crucial role as a biological tool in enhancing plant resilience to drought alongside promoting phenotypic plasticity through the establishment of mutualistic associations with the host plant species. It is now acknowledged that a combination of physical, nutritional, physiological, and cellular processes results in AM symbiosis’s contribution to plants’ ability to withstand drought (Zobel and Öpik, 2014). This mini review delves into the role of AMF in helping plants cope with drought stress, both in model plants and agriculturally important species, using a range of mechanisms involving antioxidant defenses, phytohormone mediations, osmotic adjustments, proline expressions, fungal water absorption and transport, morphological modifications, and photosynthesis (**Figure 1**). Understanding these mechanisms would be beneficial for agricultural productivity under anticipated future drought conditions.

## 2 Drought stress tolerance at biochemical level

### 2.1 Antioxidant defense mechanisms

Oxidative damage and drought stress are closely intertwined. Plants undergo an elevation in reactive oxygen species (ROS) like superoxide anion free radical (O_2_–), hydrogen peroxide (H_2_O_2_), and hydroxyl radical (OH·) among others, as well as their buildup caused by drought stress (Tiwari et al., 2017; Hasanuzzaman et al., 2021; Li et al., 2022). Drought stress can lead to an overabundance of reactive oxygen species (ROS), which can trigger an “oxidative burst” and oxidative damage in plants (Li et al., 2022). This eventually results in the structural damage of essential biomolecules leading to membrane damage and subsequent cell death in plants (Fobert and Després, 2005; Rhoads et al., 2006; Gill and Tuteja, 2010; Demidchik, 2015; Bahadur et al., 2019). AMF’s generation of ROS is a well-studied phenomenon (Fester & Hause, 2005; Zou et al., 2021) and is essential to the process of fungal colonization. The early colonization of roots by AM fungus is largely dependent on the formation of hydrogen peroxide in the cortical cells containing mycorrhiza. Nevertheless, this production is quickly eliminated by enzymes like superoxide dismutase (SOD), catalase (CAT), and carotenoids (Kapoor and Singh, 2017; Zou et al., 2021). An optimal ROS level is crucial for molecular signaling in plant growth, development, adaptation, and response to different abiotic and biotic stresses (Liu and He, 2016; Mittler, 2017; Li et al., 2022). Thus, maintaining a balance between ROS generation and ROS scavenging in stressful environments is crucial for the survival of plants (Li et al., 2022). AMF mitigates oxidative damage and enhances drought tolerance through two distinct strategies. The first strategy entails the absorption of water using hyphae followed by its subsequent transfer to the host. This process enhances the water content and reduces the production of ROS (Huang et al., 2017; Bahadur et al., 2019). The second strategy involves an increase in the generation of diverse antioxidants through a symbiotic relationship (Abbaspour et al., 2012; Bahmani et al., 2018; Bahadur et al., 2019). Heat shock transcription factors (*Hsfs*) play a crucial role in signal transduction and gene response to stress (Si et al., 2021; Ma et al., 2022). In addition, certain members of the Hsfs, including *SPL7*, *HsfA1b*, *HsfA4a* and *HsfA8*, play a role in maintaining the balance of reactive oxygen species (ROS) during drought stressed conditions (Hoang et al., 2019). Furthermore, it is worth noting that *Hsfs* possess the ability to detect ROS in plant cells. These *Hsfs* play a crucial role in regulating the oxidative burst during times of stress (Miller et al., 2008). AMF has the potential to activate antioxidant defense systems and enhance *Hsfs* transcription levels, thereby mitigating the oxidative damage induced by drought stress. *Diversispora spurca* enhances the expression of *JrHsf03*, *JrHsf22*, and *JrHsf24* in drought stressed *Juglans regia* (walnut), helping to alleviate the effects of drought stress (Ma et al., 2022). Ascorbate plays a crucial role in eliminating H_2_O_2_ through the action of ascorbate peroxidases that utilize ascorbate as an electron donor (Foyer and Noctor, 2011; Bárzana et al., 2015). A recent study highlighted the ascorbate buildup during drought. This process scavenges H_2_O_2_, as its concentration decreases significantly compared to well-watered conditions. Results also indicate that the activities of catalase (CAT), glutathione reductase (GR), guaiacol peroxidase (G-POD), and ascorbate peroxidase (APX) had a more positive impact on drought recovery in citrus plants that had been inoculated with AMF compared to non-AMF inoculated plants (Wu et al., 2006a, Wu et al., 2006b; Wu et al., 2006c; Wu et al., 2007b, Wu et al., 2013) (**Table 1**). In a related study on the inoculation of *Diversispora spurca* in *Juglans regia*, it was found that mycorrhizal plants exhibited increased peroxidase, catalase, and superoxide dismutase activities compared to non-mycorrhizal plants during periods of drought (Ma et al., 2022). The production of reactive oxygen species (ROS) is caused by respiratory burst oxidase homologs (*Rbohs*), a NADPH oxidase that also regulates a wide variety of biological processes related to biotic and abiotic stressors, including plant responses to drought (Sagi and Fluhr, 2006; Chapman et al., 2019; Tarawneh et al., 2020; Li et al., 2022; Zhang et al., 2022). AMF significantly reduced the expression of several *Rbohs* genes in drought stressed *Bombax ceiba* seedlings (Ma et al., 2022). In seedlings, *Rbohs* were slightly upregulated by AMF for well-water (WW) treatment (Ma et al., 2022). Additional studies have indicated that the symbiotic relationship between AMF and plants results in higher transcription levels of enzymatic antioxidants and components involved in the biosynthesis of ascorbate and glutathione (Marulanda et al., 2007; Abbaspour et al., 2012; Bahadur et al., 2019). This suggests a sophisticated transcriptional regulation of the antioxidant system. Additional investigations are needed to investigate the notable interplay between fungal symbiosis with plants and antioxidant systems.

**Table 1:** Mechanisms of drought stress tolerance through fungal symbiosis in plants.

Stress tolerance at Biochemical Level		
Specific Mechanisms	Representative examples	References
**Antioxidant mechanisms** (Generation of diverse antioxidants through a symbiotic relationship thereby mitigating drought stress)	**Ascorbate** eliminates H_2_O_2_ through the action of **ascorbate peroxidases** thereby aiding drought tolerance in plants during symbiotic association.	Foyer and Noctor, 2011 **;** Bárzana et al., 2015 **).**
	Enhanced activities of **catalase (CAT), glutathione reductase (GR), guaiacol peroxidase (G-POD),** and **ascorbate peroxidase (APX)** in citrus plants that had been inoculated with AMF have been reported.	Wu et al., 2006a; Wu et al., 2006b, Wu et al., 2006c; Wu et al., 2007b, Wu et al., 2013.
**Phytohormone mediated mechanisms** (Enhanced ability of mycorrhizal plants to tolerate water-stressed conditions are associated with alterations in hormonal regulation)	Enhanced production of **Abscisic acid (ABA), the abiotic stress hormone** in AMF host plants thereby improving the plant’s ability to withstand drought conditions.	Calvo-Polanco et al., 2013 **;** Hong et al., 2013 **;** Martín-Rodríguez et al., 2016 **;** Bahadur et al., 2019.
	Rise in the endogenous concentrations of **Jasmonic Acid (JA),** its precursor 12-oxophytodienoic acid, as well as derivatives such as 11-hydroxy jasmonic acid and 12-hydroxy jasmonic acid in *Digitaria eriantha* inoculated with AMF after exposure to drought and salinity stress.	López-Ráez, 2016.
	**Strigolactones (SLs)** are phytohormones derived from carotenoids and are secreted by plants. SLs have been found to mitigate the negative impacts of drought stress through the regulation of plant physiological processes during Arbuscular mycorrhizal fungus (AMF) symbiosis.	Ruiz-Lozano et al., 2016.
**Proline Mediated Mechanisms** (Proline produced by plants serves as an osmoprotectant in response to drought stress. It maintains the cellular osmotic balance in plants thereby alleviating the negative effects of drought stress)	AMF colonization of plant roots leads to **proline** accumulation under water-limited conditions. Accumulation of proline in plants was observed to be associated with the drought resistance induced by AMF symbiosis with proline serving as an osmoprotectant.	Ruiz-Lozano and Azcón, 1995 **;** Azcon et al., 1996 **;** Goicoechea et al., 1998 **;** Yooyongwech et al., 2013 **;** Rapparini and Peñuelas, 2014 **;** Goicoechea et al., 1998.
Stress tolerance through Water Absorption and Transport		
**Regulation through Aquaporins** (AMF symbiosis regulates various aquaporins within the host plant during drought stress)	Tomato plants infected with AMF showed an increase in the ability of water transport through the roots of AMF. This can be attributed to the overexpression of ***LeNIP3;1*, which encodes for NOD26-like intrinsic proteins (NIP).** AMF colonization induced the expression of certain plant genes encoding AQPs, such as ***RpPIP2;1*** in *Robinia pseudoacacia.* This induction serves as a mechanism to enhance the flow of water to particular plant tissues during periods of drought.	Chitarra et al., 2016. He et al., 2016 **;** Bahadur et al., 2019.
**Regulation through Osmotic adjustment** (Osmotic adjustment aids plants in maintaining a water potential gradient, facilitating the movement of water from the soil into the roots)	Inoculation of AMF can enhance the drought stress tolerance of citrus plants by improving **osmotic adjustment.** The growth performance and **osmotic adjustment** in *Macadamia tetraphylla L.* were improved by forming a symbiotic relationship with AMF through the buildup of soluble sugar, proline, and free amino acids in response to drought stress.	Kubikova et al., 2001 **;** Wu and Xia, 2006b **;** Wu et al., 2007a, Wu et al., 2013 **;** Abbaspour et al., 2012. Yooyongwech et al., 2013
**Regulation through Stomatal Aperture** (The role of stomatal architecture in host plants has been extensively regulated during AMF symbiosis under drought conditions)	Mycorrhizal symbiosis impacts the **stomatal density** in plants inoculated with *R.intraradices* in water-stressed conditions. High stomatal density enhances a plant’s ability to absorb CO_2._	Chitarra et al., 2016
Stress Tolerance through Morphological Modifications		
**Regulation through root system architecture** (Root System Architecture (RSA), organization of roots within the soil that plays a significant role in a plant’s ability to withstand under adverse soil conditions)	Drought stress restricts the effectiveness of **RSA** in trifoliate orange seedlings. Inoculation with *G. mosseae* resulted in higher active and total absorption regions of the root structures thereby mitigating drought stress.	Wu and Xia, 2006a
**Regulation through extraradical hyphae** (Extraradical hyphae, with a diameter of 2-5 μm, penetrate through soil pores, typically inaccessible to root hairs)	Movement of water through **mycorrhizal extraradical hyphae** results in the apoplastic water flow within plant roots.	Bárzana et al., 2012
Stress Tolerance through through Photosynthesis		
**Regulation through Photosynthesis** (AMF plants in comparison to non-AMF plants exhibit less damage to their photosynthesis machinery under drought stress)	AMF plants exhibit improved photosystem II efficiency during episodes of drought stress in addition to increased transpiration rates following drought recovery.	Germ et al., 2005 **;** Ruiz-Sánchez et al., 2010

### 2.2 Phytohormone-mediated mechanisms

The enhanced ability of mycorrhizal plants to tolerate water-stressed conditions is associated with alterations in hormonal regulation, specifically in Abscisic acid (ABA) signaling (López-Ráez, 2016) (**Table 1**). ABA has been shown to play a crucial role in arbuscular development during AMF symbiosis (Herrera-Medina et al., 2007; Martín-Rodríguez et al., 2011; López-Ráez, 2016). Several investigations have yielded valuable insights into the mechanisms behind the enhanced production of ABA in AMF host plants. This increased ABA production plays a crucial role in improving the plant’s ability to withstand drought conditions (Calvo-Polanco et al., 2013; Hong et al., 2013; Martín-Rodríguez et al., 2016; Bahadur et al., 2019). The fungus regulates ABA content in host roots during drought (Estrada-Luna and Davies, 2003; Aroca et al., 2008; López-Ráez, 2016). A study by (Calvo-Polanco et al., 2013) found that mycorrhizal plants exposed to severe stress showed a significant rise in ABA levels, also known as the ‘abiotic stress hormone’. This rise is associated with priming, which enhances the plant’s ability to tolerate stress. ABA is an essential factor for the successful establishment and functioning of AMF symbiosis. It plays a crucial role in regulating arbuscular development (Herrera-Medina et al., 2007; Martín-Rodríguez et al., 2011; Pozo et al., 2015; López-Ráez, 2016). Researchers also observed a concurrent upregulation of two plant genes, *D-myo-inositol-3-phosphate synthase*, and *14-3-3-like protein GF14*, involved in ABA signaling transduction, indicating their involvement in the synergistic effects of the symbiotic partners to improve the plant’s resistance to drought (Li et al., 2016; Bahadur et al., 2019).

Jasmonic acid (JA) and its derivatives, known as jasmonates, are believed to play a crucial role in AMF symbiosis (López-Ráez, 2016) (**Table 1**). Reports suggests that there is an observed rise in the endogenous concentrations of JA, its precursor 12-oxophytodienoic acid, as well as derivatives such as 11-hydroxy jasmonic acid and 12-hydroxy jasmonic acid in *Digitaria eriantha* plants inoculated with AMF after exposure to drought and salinity stress (López-Ráez, 2016; Pedranzani et al., 2016)). Previous research by (Sánchez-Romera et al., 2016) has demonstrated that AMF symbiosis along with the application of exogenous methyl jasmonate can mitigate the negative impact of drought on root hydraulic conductivity within common bean plants (López-Ráez, 2016). It has been suggested that the observed protection might be linked to a decrease in salicylic acid (SA) amounts due to a negative interaction between JA and SA (Pieterse et al., 2009; López-Ráez, 2016; Sánchez-Romera et al., 2016).

Strigolactones (SLs) are phytohormones derived from carotenoids and are secreted by plants. During the pre-contact phase, labile signaling molecules are released to attract AMF and help them identify a nearby host. AMF induces oxidative metabolism upon detecting SLs, leading to enhanced hyphal branching and growth. This promotes physical contact with the roots of a host plant, ultimately driving symbiotic association (Kretzschmar et al., 2012; Mori et al., 2016; Pandey et al., 2016; Bahadur et al., 2019) (**Table 1**). *Rhizophagus irregularis* has been found to stimulate the biosynthesis of SLs within lettuce and tomato plants during periods of drought, suggesting that AMF symbiosis promotes SL production (Ruiz-Lozano et al., 2016). The study demonstrated that the expression of the *SlCCD7* gene, which is responsible for the production of SLs in tomatoes, was significantly increased during drought stress in the host roots (Ruiz-Lozano et al., 2016). ABA and SLs share a common biosynthetic origin as apocarotenoids and are classified as “stress hormones.” In mycorrhizal plants under stress conditions, a positive correlation between ABA-SLs was also observed (Aroca et al., 2013; López-Ráez, 2016; Ruiz-Lozano et al., 2016). The interplay between AM symbiosis and strigolactones have been found to mitigate the adverse impacts of drought through their regulation of plant physiological processes.

### 2.3 Proline mediated mechanisms

Proline serves as an osmoprotectant, which is produced by plants as a response to stress caused by drought. Maintaining cellular osmotic balance is beneficial in alleviating the negative effects of drought stress (Koyro et al., 2012; Bahadur et al., 2019) (**Table 1**). Additional evidence supports the significant role of proline in osmoregulation and the scavenging of free radicals (Yooyongwech et al., 2013). Moreover, it serves as a molecular chaperone, aiding in the stabilization of subcellular structures thereby safeguarding plant cells from the detrimental impacts of drought stress (de Carvalho et al., 2013; Yooyongwech et al., 2013). Studies pointed out that AMF colonization of plant roots leads to proline accumulation under water-limited conditions (Ruiz-Lozano and Azcón, 1995; Azcon et al., 1996; Goicoechea et al., 1998; Yooyongwech et al., 2013; Rapparini and Peñuelas, 2014). The increased buildup of proline observed in these experiments was found to be associated with the drought resistance induced by AMF symbiosis, with proline serving as an osmoprotectant. The colonization of *Medicago sativa L.* roots by AMF leads to the accumulation of proline in both roots and under water stress conditions (Goicoechea et al., 1998). During the symbiosis of AMF in tomato plants (*Solanum lycopersicum*), the level of proline concentrations showed an extensive rise in response to water stress (WS) (Chitarra et al., 2016).

## 3 Drought stress tolerance through water absorption and transport

### 3.1 Regulation through aquaporins

Aquaporins (AQPs) are a group of integral membrane proteins that have an essential role in facilitating the transportation of water across cell membranes (Chitarra et al., 2016). AMF symbiosis regulates various aquaporins within the host plant, which include those from different subfamilies (**Table 1**). The mechanism of AQP gene regulation is influenced by watering conditions as well as the extent of drought stress. Certain aquaporins can transport both water and other physiologically important molecules, thereby contributing to the performance of the host plant (Bárzana et al., 2014). During drought stress, the upregulation of two AQP genes, *GintAQPF1* and *GintAQPF2*, was observed in the extraradical mycelia of *R. irregularis* and mycorrhizal roots. This finding suggests that AMF plays a direct role in enhancing the resilience of plants to water deprivation (Ying-Ning et al., 2013; Li et al., 2013a). In a separate study, tomato plants infected with AMF showed an increase in the ability of water transport through the roots of AMF. This increase was found to be associated with the overexpression of a gene called *LeNIP3;1*, which encodes for NOD26-like intrinsic proteins (NIP) (Chitarra et al., 2016). AMF colonization induced the expression of certain plant genes encoding AQPs, such as *RpPIP2;1* in *Robinia pseudoacacia* (He et al., 2016; Bahadur et al., 2019). This induction may serve as a mechanism to enhance the flow of water to particular plant tissues, which is crucial for the survival of the host plant during periods of drought-related stress. In contrast, the expression of the *GintAQP1* gene in lettuce roots was found to be downregulated under conditions of water deficit, despite the improvement in root AMF (Aroca et al., 2007). In their study (Bárzana et al., 2014), offered new insights into the regulation of aquaporins in maize plants during drought stress, specifically in the context of AMF symbiosis. They found that under short-term drought-stressed conditions, AMF plants showed higher sap flow rate (Jv) and osmotic root hydraulic conductance (Lo) values compared to non-AMF plants. This can be attributed to the fact that the expression levels of several PIP proteins (ZmPIP1;1, ZmPIP1;2, ZmPIP1;3, ZmPIP1;4, ZmPIP1;6, ZmPIP2;2, and ZmPIP2;4) remained high or even increased. During prolonged periods of drought, the availability of soil water resources is reduced significantly, resulting in a decrease in both Jv and Lo values in AMF plants. In that scenario, AMF has been observed to downregulate several PIP genes, including *ZmPIP1;1*, *ZmPIP1;3*, *ZmPIP1;4*, *ZmPIP2;2*, and *ZmPIP2;4*, in both well-watered and sustained drought situations. This observed downregulation might serve as an approach to prevent water loss (Porcel et al., 2006). AM fungal aquaporins may also contribute to drought tolerance during the event of AMF symbiosis (Aroca et al., 2009; Li et al., 2013a, Li et al., 2013b). AMF aquaporins are known to play a role in facilitating water movement in both the extraradical mycelium and periarbuscular membrane (Li et al., 2013a). The increased Lo values observed in AMF plants during short-term drought stress and the elevated hydrostatic root hydraulic conductance (Lh) values observed during prolonged drought can be attributed to the functioning of fungal aquaporins (Bárzana et al., 2014).

### 3.2 Regulation through osmotic adjustment

Osmotic adjustment (OA) is considered an effective way to promote drought tolerance in plants (Wu et al., 2013). OA aids plants in maintaining a water potential gradient, facilitating the movement of water from the soil into the roots (Yooyongwech et al., 2013; Zhang et al., 2016; Bahadur et al., 2019) (**Table 1**). It involves the reduction of osmotic potential by accumulating low molecular weight solutes when exposed to stress (Martìnez et al., 2004; Wu et al., 2013). Organic (proline, glycinbetain, aspartic acid, protein, and sugars) and inorganic solutes (K^+^, Ca^2+,^ and Mg^2+^) function as osmoprotectants, aiding in water absorption and stabilizing macromolecular frameworks and subcellular membranes during dehydration stress (Gomes et al., 2010; Wu et al., 2013). The growth performance and osmotic adjustment in *Macadamia tetraphylla L.* were improved by forming a symbiotic relationship with AMF. This improvement was achieved through a buildup of various compounds, including soluble sugar, proline, and free amino acids, in response to drought conditions (Yooyongwech et al., 2013). Multiple studies have shown that the inoculation of AMF can enhance the drought stress tolerance of citrus plants by improving osmotic adjustment (OA) (Kubikova et al., 2001; Wu and Xia, 2006b; Wu et al., 2007a; Abbaspour et al., 2012; Wu et al., 2013).

### 3.3 Regulation through stomatal aperture

The role of stomatal architecture has been extensively studied during AMF symbiosis in response to the water-stressed condition in *Solanum lycopersicum* (tomato plants) (Chitarra et al., 2016). The study quantified the stomatal density in mature leaves of AMF and control NS plants. The findings showed that mycorrhizal symbiosis has an impact on stomatal density, particularly in plants inoculated with *R. intraradices*. The density of stomatal cells in this condition was approximately double compared to that of the control and plants inoculated with *F. mosseae*. A high stomatal density enhances a plant’s ability to absorb CO_2_ (Chitarra et al., 2016) (**Table 1**). The study quantified *LeEPFL9* transcripts, which have an effect in regulating stomatal development, alongside the genes encoding *EPF1* and *EPF2* that act as antagonists of *LeEPFL9* thereby negatively regulating stomatal development. In tomato leaves undergoing development, the expression of these genes was observed to be significant only when AMF symbiosis was present. The steady-state levels of *LeEPFL9* transcripts showed a strong positive correlation in accordance with the higher stomatal density observed in plants colonized by *R. intraradices* (Chitarra et al., 2016).

## 4 Drought stress tolerance through morphological modifications

### 4.1 Regulation through root system architecture

Root system architecture (RSA) refers to the organization of roots within the soil particularly playing a significant role in a plant’s ability to withstand adverse soil conditions (de Dorlodot et al., 2007; Wu et al., 2013). AMF colonization can cause RSA modifications to host plants, which are influenced by factors such as plant and fungal species or genotypes, in addition to both water and nutrient availability (Wu et al., 2012; Chatzistathis et al., 2013; Wu et al., 2013; Ying-Ning et al., 2013) (**Table 1**). One report suggests that drought stress greatly restricted the effectiveness of RSA in trifoliate orange seedlings. However, inoculation with *G. mosseae* successfully mitigated this limitation and resulted in higher active and total absorption regions of the root structures. This effect was observed in seedlings grown under different soil water content levels (20%, 16%, and 12%) contrasted to those that were not inoculated with AMF (Wu and Xia, 2006a). Studies by (Orfanoudakis et al., 2010) suggest that the combined inoculation of AMF and Frankia resulted in a bigger spike in root branching in plants, specifically *Alnus glutinosa*. The alterations in RSA caused by AMF can be attributed to multiple factors such as an altered balance of cytokinin to gibberellin, improved nutritional condition in AMF plants, and the tightly controlled metabolism of endogenous polyamines (Berta et al., 1993; Wu et al., 2012; Chatzistathis et al., 2013; Wu et al., 2013).

### 4.2 Regulation through extraradical hyphae

In dry soil conditions as opposed to wet soil conditions, the hyphal water transfer may play a greater role. The movement of water through mycorrhizal hyphae plays a role in the apoplastic water flow within roots (Bárzana et al., 2012) (**Table 1**). Extraradical hyphae, with a diameter of 2-5 μm, penetrate through soil pores that are typically inaccessible to root hairs (Gianinazzi et al., 1994; Khan, 2003). K^+^ is essential for water movement by mycorrhizal hyphae. The presence of additional K^+^ simply enhanced root hydraulic conductivity in AMF plants, compared to non-AMF plants, irrespective of water conditions (El-Mesbahi et al., 2012). The mycorrhizal association is crucial in aiding the absorption of mineral nutrients, particularly those that have limited movement within the soil, like phosphorus (P), zinc (Zn), and copper (Cu) (Srivastava et al., 2002; Smith and Smith, 2011).

## 5 Regulation through photosynthesis

The symbiotic relationship between AMF and *Oryza sativa* (Rice) plants improved the efficiency of photosynthesis by more than 40% during stress conditions (Ruiz-Sánchez et al., 2010). AMF plants demonstrated improved photosystem II efficiency under drought stress in addition to increased transpiration rates following drought recovery (**Table 1**). Reports already suggest that AMF plants exhibit higher photosynthetic efficiency indicating less damage to their photosynthesis machinery under drought stress (Germ et al., 2005; Ruiz-Sánchez et al., 2010). The two combined effects probably contributed to the improved plant growth of AMF plants through improved CO_2_ fixation during and after periods of drought stress (Ruiz-Sánchez et al., 2010). Furthermore, the improved efficiency of photosystem II together with increased transpiration in AMF plants may have resulted in reduced photorespiration and subsequently reduced levels of ROS in these plants (Cadenas, 1989).

## 6 Conclusions

Current and future drought events are a serious cause of concern. As a scientific community, we must be prepared to mitigate drought events through natural and organic efforts. We anticipate heavy losses to plants and agricultural productivity due to the disturbances. AMF helps plants withstand environmental constraints, particularly drought, thereby enhancing their resilience. We discussed how AMF could protect plants at biochemical level through antioxidant defense mechanisms, phytohormone and proline-mediated mechanisms. AMF also aids in drought stress tolerance through water absorption and transport using aquaporins, making osmotic adjustments, and also through photosynthesis. Moreover, morphological modifications in plants and AMF can also contribute to the drought stress tolerance. We believe this knowledge would help fathom the ways fungal interaction with plants is useful in tolerating extreme situations.

## Author contributions

SD: Conceptualization, Investigation, Software, Visualization, Writing – original draft, Writing – review & editing. SS: Conceptualization, Investigation, Resources, Software, Visualization, Writing – original draft, Writing – review & editing.
